# Cellular oxidative stress response mediates radiosensitivity in Fus1-deficient mice

**DOI:** 10.1038/cddis.2014.593

**Published:** 2015-02-19

**Authors:** E M Yazlovitskaya, P A Voziyan, T Manavalan, W G Yarbrough, A V Ivanova

**Affiliations:** 1 Department of Medicine, Vanderbilt University School of Medicine, Nashville, TN, USA; 2 Vanderbilt-Ingram Cancer Center, Vanderbilt University Medical Center, Nashville, TN, USA; 3 Department of Surgery, Division Otolaryngology, Yale School of Medicine, New Haven, CT, USA

## Abstract

Mechanism of radiosensitivity of normal tissues, a key factor in determining the toxic side effects of cancer radiotherapy, is not fully understood. We recently demonstrated that deficiency of mitochondrial tumor suppressor, Fus1, increases radiosensitivity at the organismal, tissue and cellular levels. Since Fus1-deficient mice and cells exhibit high levels of oxidative stress, we hypothesized that dysregulation of cellular antioxidant defenses may contribute to the increased radiosensitivity. To address this potential mechanism, we treated the Fus1 KO mice with an inhibitor of pathogenic oxidative reactions, pyridoxamine (PM). Treatment with PM ameliorated IR-induced damage to GI epithelium of Fus1 KO mice and significantly increased the survival of irradiated mice. In cultured Fus1 KO epithelial cells, IR-induced oxidative stress was enhanced because of inadequate cellular antioxidant defenses, such as low levels and/or activities of cytochrome C, Sod 2 and STAT3. This resulted in dysregulation of IR-induced DNA-damage response and DNA synthesis. Treatment of Fus1 KO cells with PM or Sod 2 mimetic Tempol normalized the oxidative stress response, thus compensating to a significant degree for inadequate antioxidant response. Our findings using Fus1 KO radiosensitive mice suggest that radiosensitivity is mediated via dysregulation of antioxidant response and defective redox homeostasis.

Normal tissue sensitivity to ionizing radiation (IR) limits the therapeutic dose that can be delivered to the tumor and is responsible for early and late side effects. Minimizing the toxic effects of IR in normal cells would significantly alleviate the side effects and improve the outcome of radiotherapy. However, mechanisms regulating the response of normal tissues to IR are still not completely understood. Ionizing radiation is a strong inducer of reactive oxygen species (ROS) that are produced mostly by mitochondria.^1^ Overproduction of ROS at the time of irradiation causes mitochondrial dysfunction followed by an oxidative damage to mitochondrial DNA and proteins.^2^ It has also been suggested that ROS generated by mitochondria contribute to genomic instability after radiation exposure.^2^

We previously demonstrated that mitochondrial tumor suppressor Fus1 can modulate radiosensitivity of normal gastrointestinal (GI) tract epithelium,^3^ one of the primary targets of ionizing radiation during whole body exposure or pelvic radiotherapy.^4, 5, 6^ Given that Fus1 is involved in the regulation of mitochondrial homeostasis including generation of ROS^7, 8^ and that levels of Fus1 expression vary significantly in individual humans,^9, 10^ these findings may provide mechanistic insights into IR toxicity in normal tissues. Moreover, expression levels of Fus1 may predict individual susceptibility to radiation toxicity. To determine the mechanisms whereby Fus1 modulates cellular radiosensitivity, we utilized Fus1 KO mouse model and inhibitors of oxidative pathways with different mechanisms of actions, pyridoxamine (PM) and Tempol.

Fus1 KO mice are characterized by mitochondrial dysfunction and elevated oxidative stress.^8, 11^ Upon whole body irradiation (WBI), these mice exhibit increased mortality because of GI toxicity driven by accelerated apoptosis and untimely re-entry into cell cycle leading to GI crypt epithelial cell death and diminished crypt regeneration.^3^ Suggested causative mechanisms include dysregulation of cell cycle, apoptotic signaling, DNA repair and oxidative stress response.^3^ Since ROS generation occurs immediately upon irradiation^12^ and high ROS levels and oxidative stress are the main features of mitochondrial dysfunction in Fus1 KO mice,^8, 11^ we hypothesized that amelioration of oxidative stress may abrogate increased radiosensitivity of Fus1 KO tissues, thus improving crypt regeneration and animal survival.

Pyridoxamine treatment ameliorates pathogenic oxidative pathways in diabetes and other pro-oxidative stress conditions, including following exposure to ionizing radiation.^13, 14^ PM has been shown to scavenge and inhibit the production of toxic ROS and carbonyl species, which also are major damaging factors in irradiated biological tissues.^14^ Tempol is a cell membrane-permeable amphilite that dismutates superoxide catalytically and facilitates hydrogen peroxide metabolism by catalase-like actions.^15^

In the present study, we demonstrated that treatment with PM ameliorated IR-induced damage to GI epithelium of Fus1 KO mice and significantly increased the survival of these mice upon irradiation. Cell culture experiments showed that loss of Fus1 protein enhanced IR-induced oxidative stress because of inadequate cellular antioxidant defenses. This resulted in dysregulation of IR-induced DNA-damage response and DNA synthesis. Treatment of Fus1 KO cells with PM or Tempol normalized the oxidative stress response, thus compensating to a significant degree for the lack of Fus1. These data suggest that mechanisms of radiosensitivity can be determined by dysregulation of antioxidant response and defective redox homeostasis.

## Results

### Pyridoxamine treatment improves survival of irradiated mice

**Figure 1:**
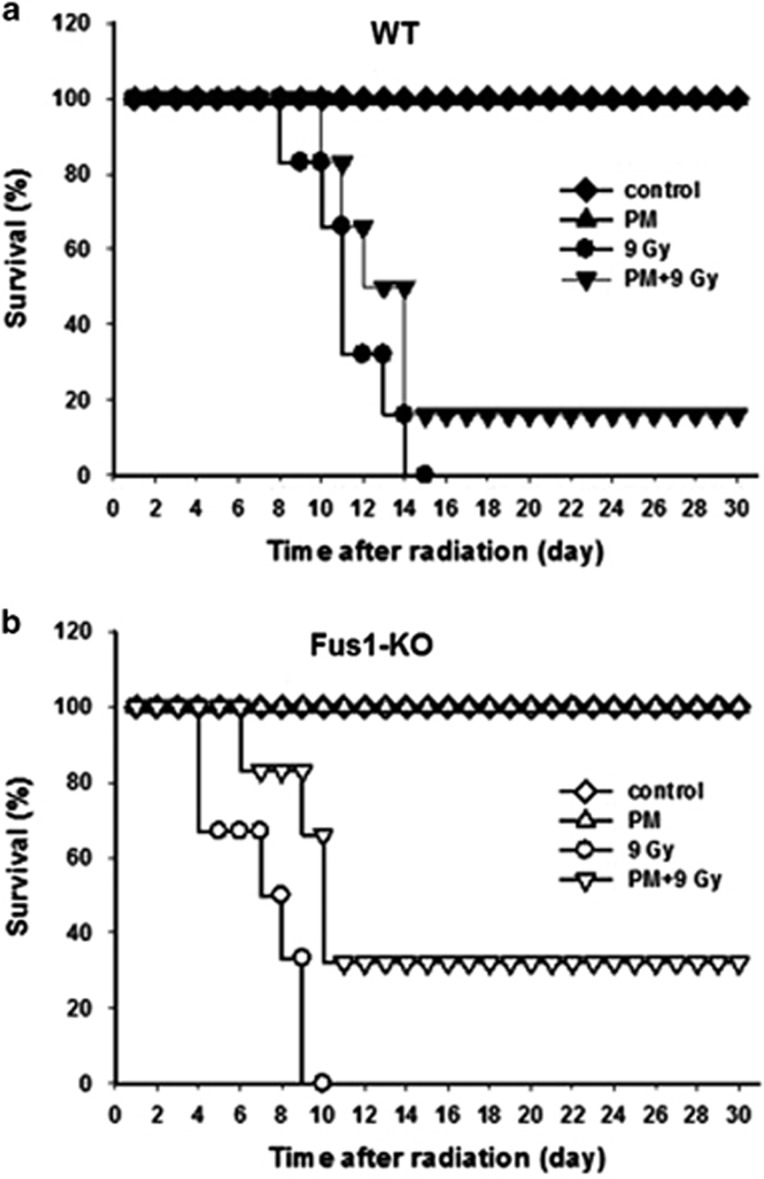
Effect of PM on survival of WT and Fus1 KO mice after WBI. WT (**a**) and Fus1 KO (**b**) female mice (5–6 weeks of age, six animals per group) were given 150 mg/kg/day PM in drinking water (triangles) or water (diamonds, circles) starting 24 h prior to exposure to 9 Gy WBI (circles, triangles down). Mice were observed for the signs of premorbid state as described in Materials and Methods. Shown are Kaplan–Meyer survival curves

We previously demonstrated that upon WBI, survival of Fus1 KO mice was decreased compared with WT mice, with the greatest difference observed at a dose of 9 Gy.^3^ Here we show that treatment of either WT or Fus1 KO mice with PM prior to 9 Gy WBI resulted in improved survival compared to mice treated with WBI alone (Figure 1). Remarkably, radioprotective effect of PM was significantly greater in Fus1 KO compared with WT mice, effectively making Fus1 KO mice more radioresistant than WT mice (Figure 1; PM+9 Gy *versus* 9 Gy: Fus1 KO mice: median survival 32%, *P*=0.0015; WT mice: median survival 16%, *P*=0.05). In particular, at 13–30 days after WBI survival of PM-treated irradiated Fus1 KO mice was about two-fold greater than the survival of PM-treated irradiated WT mice (Figure 1).

### PM treatment improved intestinal crypt regeneration in Fus1 KO mice

**Figure 2:**
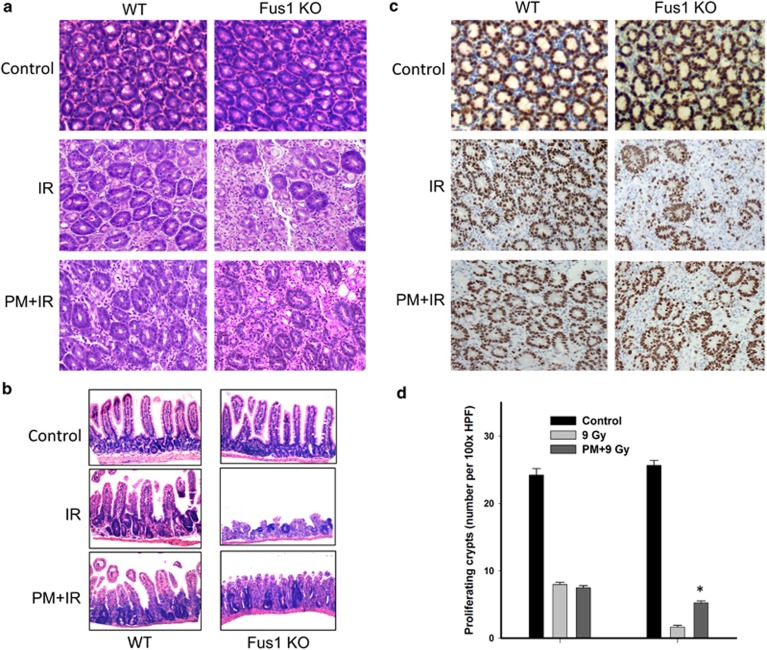
Morphology and proliferation of WT and Fus1 KO intestinal crypts after WBI**.** WT and Fus1 KO mice were treated with PM as in Figure 1 and exposed to 12 Gy WBI. Shown are micrographs of the sections of distal jejunum harvested at 96 h post IR and stained with either H&E (**a**, **b**) or with anti-BrdU antibody following BrdU injection of mice (**c**) (magnification, × 200). Shown is a bar graph of the average number of BrdU-positive proliferating crypts per HPF in each treatment group (*n*=3) with S.E.M. (**d**) *P<0.05, 9 Gy *versus* PM+9 Gy

Early intestinal damage induced by IR is a major cause of accelerated death in Fus1 KO mice.^3^ Because irradiated Fus1 KO mice showed dramatic decrease in crypt regeneration,^3^ we compared the effect of PM on crypt regeneration in WT and Fus1 KO mice (Figure 2). Staining of jejunum with H&E (Figures 2a and b) and proliferation marker KI-67 (Figure 2c) demonstrated significant improvement in crypt regeneration of PM-treated Fus1 KO mice compared with vehicle-treated Fus1 KO mice at 96 h post irradiation (Figure 2d). In contrast, crypt regeneration in irradiated PM-treated WT mice was similar to that of WT mice exposed to only WBI (Figure 2).

### Effect of IR and PM on DNA synthesis in WT and Fus1 KO epithelial cells

To further determine mechanisms of radiation damage in Fus1 KO intestine, we investigated early effects of IR with and without PM on primary cultured epithelial cells isolated from either WT or Fus1 KO mice.

**Figure 3:**
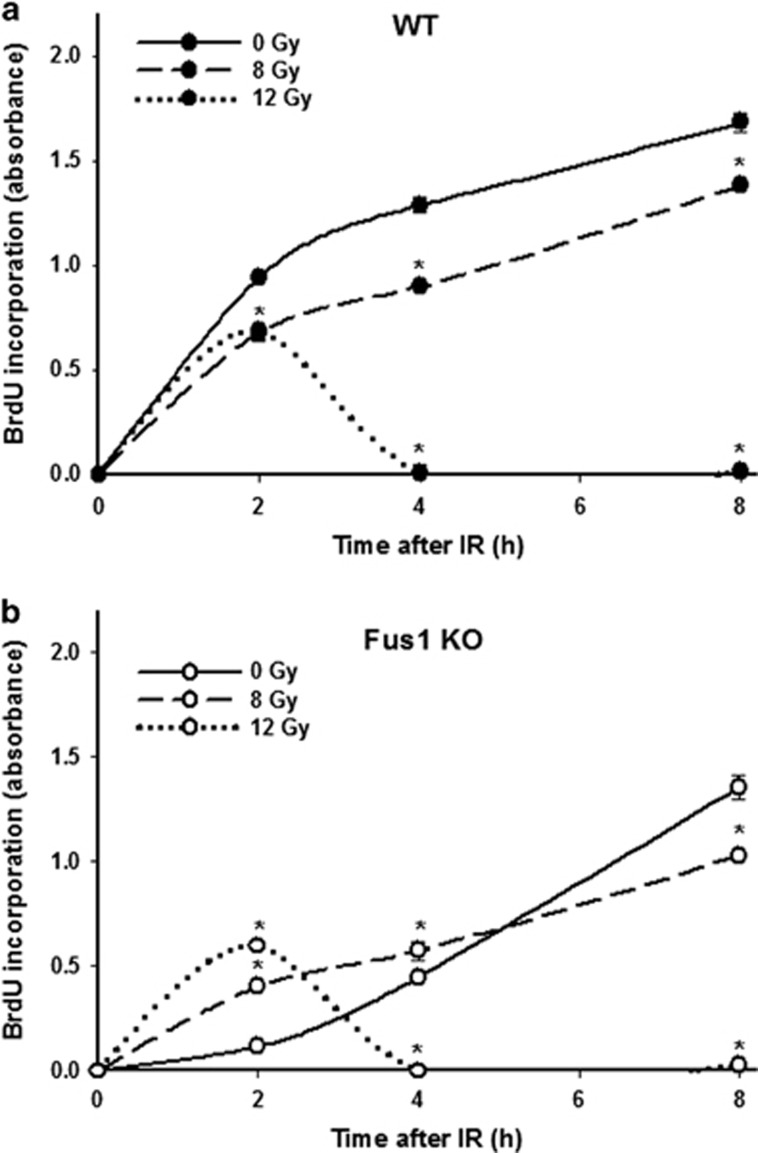
Effect of IR on DNA synthesis in WT and Fus1 KO epithelial cells**.** WT (**a**) and Fus1 KO (**b**) epithelial cells were either sham-irradiated or irradiated with 8 or 12 Gy. DNA synthesis was determined using BrdU ELISA as described in Experimental Procedures. Shown are graphs of the mean BrdU incorporation (absorbance) in each treatment group (*n*=3) with S.E.M. *P<0.05, irradiated *versus* sham-irradiated controls

First, we determined the effects of different IR doses on the rate of DNA synthesis in WT and Fus1 KO cells. Cells were sham-irradiated or irradiated with either 8 or 12 Gy, and DNA synthesis measured at 2, 4 and 8 h after IR (Figure 3). At 2 h after radiation, DNA synthesis was inhibited in irradiated WT cells compared with sham-irradiated controls (Figure 3a). In contrast, DNA synthesis in irradiated Fus1 KO cells was paradoxically increased compared with sham-irradiated controls (Figure 3b). This phenomenon was transient and was not detectable at 8 h post IR when the rates of DNA synthesis in WT and Fus1 KO cells were similar (Figure 3).

**Figure 4:**
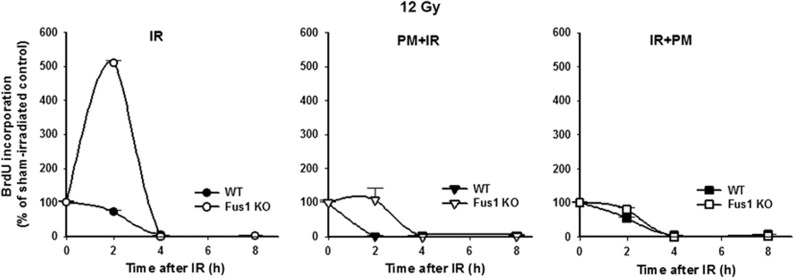
Effect of PM on DNA synthesis in WT and Fus1 KO epithelial cells after exposure to IR**.** WT (black symbols) and Fus1 KO (white symbols) epithelial cells were treated with either PBS (circles) or 1 mM PM in PBS 1 h prior to (triangles) or 5 min after (squares) 12 Gy irradiation. DNA synthesis in cells was evaluated by BrdU ELISA as described in Experimental Procedures. Shown are graphs of the mean BrdU incorporation normalized to sham-irradiated control with S.E.M. (*n*=3)

To determine the IR dose and time point that represent the most dramatic differences in Fus1 KO and WT cellular responses to IR, we normalized the changes in DNA synthesis in the irradiated cells to basal levels and compared these changes between WT and Fus1 KO cells. Since the most significant difference in IR-induced changes of DNA synthesis between WT and Fus1 KO cells occurred early following exposure to 12 Gy (Figure 4, left panel), we have further investigated radioprotective effect of PM at this IR dose. In these experiments, PM was added to cells either 1 h prior to irradiation (PM+IR) or immediately after irradiation (IR+PM). DNA synthesis was significantly increased in Fus1 KO cells compared with WT cells at 2 h post irradiation (Figure 4, left panel; 5-fold-increase), consistent with the data shown in Figure 3. Pretreatment with PM before IR resulted in abrogation of cell division in WT cells and absence of increase in DNA synthesis in Fus1 KO cells compared with WT cells at 2 h after IR (Figure 4, middle panel). Interestingly, when added to cells post IR, PM still protected from the IR-induced increase of DNA synthesis in Fus1 KO cells relative to WT cells (Figure 4, right panel). The difference in BrdU incorporation between Fus1 KO cells treated with PM before or after IR was not statistically significant. These data suggest that PM treatment normalizes DNA synthesis in Fus1 KO cells in response to IR.

**Figure 5:**
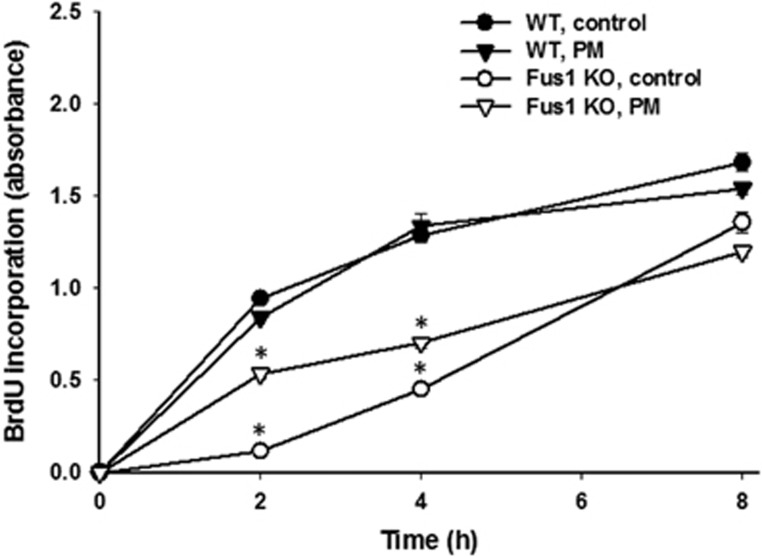
Effect of PM on DNA synthesis in WT and Fus1 KO epithelial cells. WT (black symbols) and Fus1 KO (white symbols) epithelial cells were treated with either PBS (circles) or 1 mM PM in PBS (triangles) for 1 h prior to addition of BrdU. DNA synthesis in the cells was evaluated by BrdU ELISA as described in Experimental Procedures. Shown are graphs of average BrdU incorporation (absorbance) in each treatment group (*n*=3) with S.E.M. *P<0.05, control *versus* PM treatment for Fus1 KO cells; controls WT *versus* Fus1 KO cells; and PM WT *versus* Fus1 KO cells

Indeed, the same phenomenon was observed in Fus1 KO cells even in the absence of irradiation (Figure 5). Fus1 KO cells had a slower proliferation rate compared with WT cells. PM treatment significantly increased DNA synthesis of Fus1 KO cells after 2 and 4 h of treatment, thus bringing the rate of DNA synthesis in line with that in WT cells (Figure 5b). Importantly, PM treatment did not affect the rate of DNA synthesis in WT cells (Figure 5a). To further investigate the mechanism of this phenomenon, we focused on cellular antioxidant and DNA repair machinery induction by IR.

### Pyridoxamine treatment improves antioxidant response in Fus1 KO cells

**Figure 6:**
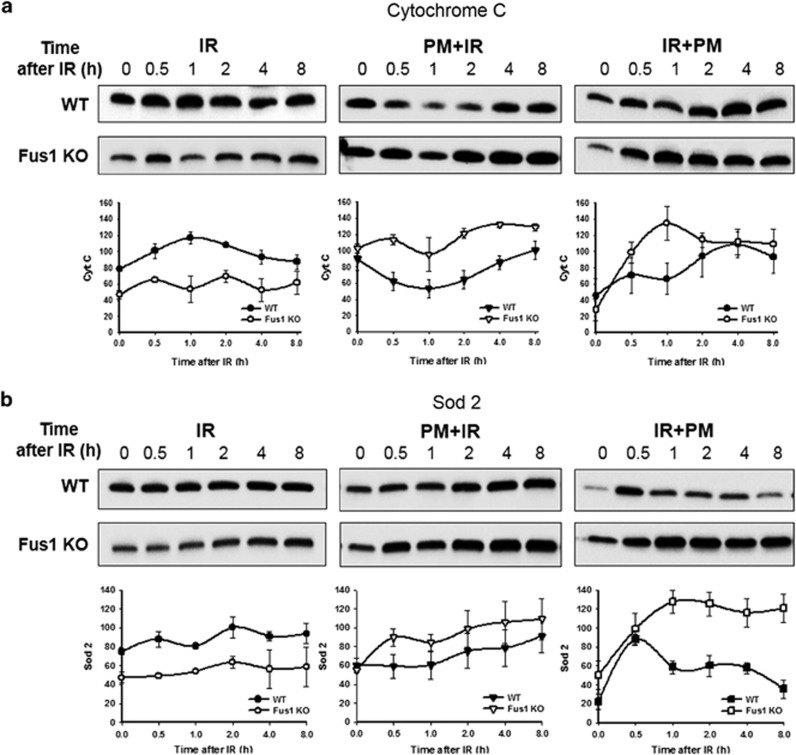

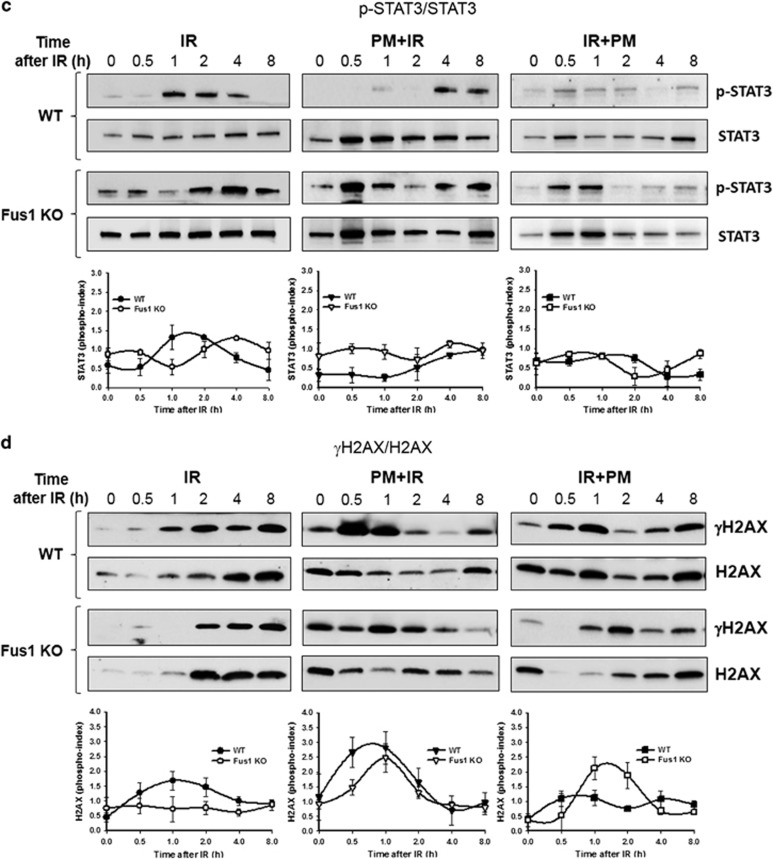
Effect of PM on activation of IR-induced antioxidant and DNA-damage responses in WT and Fus1 KO epithelial cells**.** WT (black symbols) and Fus1 KO (white symbols) epithelial cells were treated with either saline (circles) or 1 mM PM in saline 1 h prior to (triangles) or immediately after (squares) 12 Gy irradiation. Cells were lysed at 0–8 h after IR. Shown are representative western immunoblot analyses of changes in total protein levels of cytochrome C (**a**) and Sod 2 (**b**); and changes in phospho- and total protein levels of STAT3 (**c**) and H2AX (**d**). Quantitative analyses of band intensities shown below the corresponding blots were performed using ImagJ software; the values were normalized to loading control visualized by stain-free gels (Bio-Rad, Inc.) and presented as a fold change over 0 h time point. Experiments were repeated three times with similar results. Shown are the representative images and graphs of average values from the three experiments with S.E.M.

Formation of ROS occurs immediately after irradiation, continues for several hours, and results in oxidative DNA damage.^12^ The extent of DNA damage depends on the level of produced ROS and the robustness of innate antioxidant defenses. We previously found that loss of Fus1 results in increased ROS production^8, 11^ and demonstrated that Fus1 KO cells have weak antioxidant defenses based on the lower levels and slower accumulation of antioxidant proteins after IR.^3^ Based on these observations, we hypothesized that dysregulation of cellular antioxidant defenses may contribute to increased radiosensitivity. To address this potential mechanism, we analyzed antioxidant response (cytochrome C, Sod 2 and STAT3) and DNA-damage (H2AX) proteins in the irradiated cells treated with PM, an inhibitor of pathogenic oxidative reactions. In this study, Fus1 KO cells have lower basal levels of antioxidant proteins cytochrome C and Sod 2 compared with WT cells (Figures 6a and b, left panels). Although IR exposure led to increased levels of these proteins in Fus1 KO cells, they did not reach corresponding levels in WT cells (Figures 6a and b, left panels). Treatment with PM either before or after irradiation significantly increased protein levels of cytochrome C and Sod 2 in the irradiated Fus1 KO cells relative to WT cells (Figures 6a and b, middle and right panels).

Because STAT3 is involved in protection from oxidative stress through upregulation of Sod 2,^18, 19, 20^ effects of PM treatment on STAT3 activation (phosphorylation) in irradiated WT and Fus1 KO cells was explored (Figure 6c). Radiation-induced activation of STAT3 was less robust in Fus1 KO cells relative to WT cells with apparent biphasic activation in Fus1 KO cells (Figure 6c, left panel). Upon PM treatment, STAT3 activation in Fus1 KO cells was fully restored or became greater than in similarly treated WT cells (Figure 6c, middle and right panels).

### Pyridoxamine treatment potentiates activation of IR-induced response to DNA damage

Ionizing radiation causes DNA damage and activates DNA-damage response (DDR) pathways. One early marker of DDR activation is accumulation of phosphorylated H2AX (*γ*H2AX), whose levels increase immediately after IR exposure and decline over a period of hours as cells repair the damage.^1^ Irradiated Fus1 KO cells showed a dramatically delayed accumulation of *γ*H2AX at very low levels compared to a classical early robust response in WT cells (Figure 6d, left panel). Treatment with PM prior to irradiation normalized the level and kinetics of *γ*H2AX accumulation in Fus1 KO cells (Figure 6d, middle panel). Remarkably, post-IR PM treatment still resulted in a robust albeit somewhat delayed H2AX phosphorylation in Fus1 KO cells (Figure 6d, right panel).

### Effect of Tempol on DNA synthesis in irradiated WT and Fus1 KO epithelial cells

**Figure 7:**
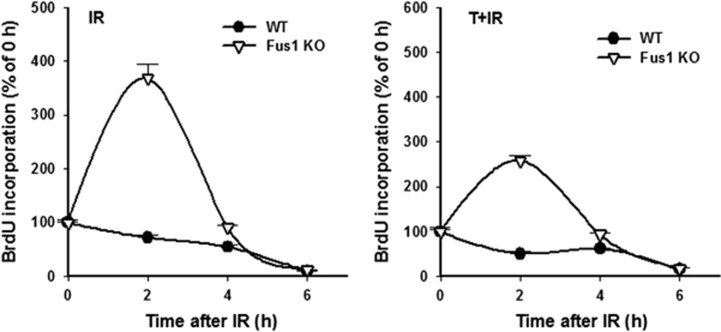
Effect of Tempol on DNA synthesis in WT and Fus1 KO epithelial cells after exposure to IR**.** WT (black circles) and Fus1 KO (white triangles) epithelial cells were treated with either PBS (left panel) or 1 mM Tempol in PBS (right panel) 1 h prior to 12 Gy irradiation. DNA synthesis in cells was evaluated by BrdU ELISA as described in Experimental Procedures. Shown are the graphs of the mean BrdU incorporation normalized to sham-irradiated control with S.E.M. (*n*=3)

To further confirm the role of oxidative reactions in mechanisms of radiosensitivity of Fus1 KO epithelial cells, we used another inhibitor of oxidative reactions, Sod 2 mimetic Tempol. In these experiments, cells were irradiated with 12 Gy (IR) or treated with 1 mM Tempol 1 h prior to radiation exposure (T+IR). DNA synthesis was significantly increased in Fus1 KO cells compared with WT cells at 2 h post irradiation (Figure 7, IR, left panel; four-fold-increase), similar to the effect observed earlier (Figure 4, IR, left panel; five-fold increase). Pretreatment with Tempol before IR resulted in significant decrease of cell division in both cell types compared with 2 h time point of IR exposure alone (Figure 7, T+IR, right panel). Interestingly, effect of Tempol on IR-induced increase in DNA synthesis in Fus1 KO cells was less pronounced in comparison with PM (Figure 4).

## Discussion

Fus1 deficiency results in mitochondrial dysfunction leading to chronic oxidative stress that affects multiple cellular functions.^7, 8, 11, 21^ Recently, we demonstrated that Fus1 KO mice exhibit a much higher radiosensitivity than WT mice.^3^ Ionizing radiation activates multiple pathways that determine the final outcome of cellular response: either DNA repair and cell survival or irreparable DNA damage, apoptosis and cell death. Several of these pathways, that is, DNA damage repair, cell cycle, apoptosis, oxidative stress response, are dysregulated in Fus1 KO intestinal epithelium after irradiation.^3^ Here, we showed that treatment with the inhibitor of oxidative reactions PM normalized cellular oxidative stress response compromised by Fus1 deficiency, thus improving regeneration of intestinal crypts and survival of irradiated Fus1 KO mice. Previously, we found that Fus1-deficient stem cells cannot repopulate crypts after IR-induced damage;^3^ therefore, data presented here indicate that PM protects Fus1 KO crypt epithelial stem cells from IR damage.

Sham-irradiated epithelial Fus1 KO cells are characterized by low rate of DNA synthesis and slow cell growth. Upon the IR damage, dysregulated antioxidant defense mechanisms in Fus1 KO cells prevent them from entering early cell growth arrest necessary for efficient DNA repair. In both sham-irradiated and irradiated Fus1 KO cells, PM treatment brought the levels of DNA synthesis and growth rate of Fus1 KO cells close to those of WT cells via normalization of antioxidant defense mechanisms. This is in line with protective effect of PM toward Sod 2 and cytochrome C as well as transcription factor STAT3 (a known activator of Sod 2^18, 19, 20^), which are key antioxidant defense proteins in early IR response.^3, 22, 23^ It is also consistent with improvement of DNA-damage response upon PM treatment, since the connection between oxidative stress and DNA-damage response is well established.^24, 25, 26, 27^

Role of oxidative reactions in mechanisms of radiosensitivity in Fus1 KO cells was further investigated by using another inhibitor of oxidative reactions, Sod 2 mimetic nitroxide Tempol which also exhibits radioprotective effect.^28, 29^ In our experiments, protective effect of Tempol on irradiated Fus1 KO cells was similar to that of PM. This confirms the importance of oxidative pathways in modulation of radiosensitivity since PM and Tempol inhibit oxidative reactions via different mechanisms of action.

Interestingly, we also observed a mitigation effect of PM treatment, that is, when PM was added to the cells shortly after irradiation. As formation of ROS may continue for several hours after irradiation,^12^ this observation may indicate the importance of secondary cascades of oxidative species production that are delayed compared with those produced directly from tissue radiolysis. It also indicates that PM does not interfere with IR directly but rather protects from IR-induced oxidative damage to macromolecules including those involved in cellular antioxidant defenses.

As we have previously shown, Fus1 expression in normal tissues is suppressed by increased ROS levels; thus, smoking, chronic exposure to environmental insults or systemic inflammation could be associated with low Fus1 levels in normal tissues.^8, 30^ Based on our current and previous findings,^8, 30^ we suggest that normal tissues of many cancer patients with lower systemic levels of Fus1 will be more sensitive to radiotherapy. Thus, Fus1 levels in tissues and blood may be predictive of individual susceptibility to the side effects of IR in normal tissues and may guide the design of individual treatment regimens. Moreover, in susceptible individuals with low Fus1 levels, PM or other antioxidative treatments may be used to protect normal tissues during radiation cancer therapy.

## Materials and Methods

### Mouse model and treatment

Fus1 KO mice on a mixed background were generated by Dr. Alla Ivanova at NCI-Frederick. The mice were extensively backcrossed to obtain a homogenous 129sv genetic background in the laboratory of Dr. S. Andersen (NCI-Frederick) and mating pairs were obtained from his laboratory.^7^ Backcrossing of Fus1 KO mice to 129sv background was done for at least 10–12 generations. Colonies of Fus1 KO and WT 129sv mice were maintained in the same facility at Vanderbilt University School of Medicine, thus eliminating a possibility of strain- and environment-specific artefacts. All animal experiments were performed according to a protocol approved by the Institutional Animal Care and Use Committee. The animals were housed under a 12 h light/darkness cycle. Food (Purina Rodent Chow, St. Louis, MO, USA) and water were provided *ad libitum*. WBI were carried out in a Mark I 137Cs irradiator (J.L. Shepherd and Associates, San Fernando, CA, USA) at a dose rate of 167 cGy/min and total dose of 9 Gy. Female mice (5–6 weeks old) were irradiated as previously described.^13, 16^ Sham-irradiated mice were handled the same way as irradiated animals with the exception of irradiation. Pyridoxamine (PM) was bought from Sigma and was given to mice in drinking water (150 mg/kg/day) 24 h prior to irradiation and PM treatment continued until the end of experiment. Experimental groups consisted of six animals.

### Evaluation of mouse survival

Mice were treated with PM or vehicle (water) and either sham-irradiated or irradiated with 9 Gy. Each experimental group included six animals. Over the course of 30 days, mice were weighed daily and observed closely for the signs of a premorbid state. These signs included hypoactivity; shallow, rapid and/or labored breathing; failure to groom; failure to respond to stimuli; hunched posture; dehydration; and weight loss according to the approved protocol. Mice were killed when the signs of premorbid state were observed. Animals without signs of premorbid state were killed at the end of experiment. Percent survival was calculated using Kaplan–Meyer analysis.

### Evaluation of proliferation in mouse small intestine

Mice were sacrificed after 96 h of treatment. The proximal jejunum and distal ileum were fixed for histology in 10% formalin in PBS and stained with H&E. For evaluation of cell proliferation, tissues were stained with anti-KI-67 antibodies and visualized with DAB.^3^

### Cell cultures and treatment

Spontaneously immortalized WT and Fus1 KO normal kidney epithelial cells obtained using ‘3T3' protocol as described in Yazlovitskaya *et al.*^3^ Both cell lines were passaged for about the same number of passages after immortalization. Cells were maintained in 10% FBS/DMEM high glucose medium. For irradiation, cells were grown to 70–80% confluency and exposed to 12 Gy IR using Mark I 137Cs irradiator. Cells were treated with 1 mM PM either 1 h prior to or immediately after irradiation. For some experiments, cells were treated with 1 mM Tempol, Sod 2 mimetic (Sigma), 1 h prior to irradiation.

### Evaluation of DNA synthesis in cell cultures

DNA synthesis was measured using incorporation of 5-bromodeoxyuridine (BrdU) and enzyme-linked immunosorbent assay-based 5-Bromo-2′-deoxy-uridine Labeling and Detection Kit III (Roche Applied Science, Penzberg, Upper Bavaria, Germany) as previously described.^17^ Experiments were performed three times in triplicates. BrdU incorporation was quantified by absorbance at 405 nm.

### Western immunoblot analysis

Treated cells were incubated for 0, 0.5, 1, 2, 4 and 8 h 37 °C and lysed using M-PER kit (Thermo Fisher Scientific, Rockford, IL, USA). Protein concentration was quantified using BCA reagent (Thermo Fisher Scientific). Protein extracts (100 *μ*g) were subjected to western immunoblot analysis. Western immunoblot analysis was performed using antibodies against the following proteins: Cytochrome C (Santa Cruz Biotechnology, Dallas, TX, USA), Sod 2 (R&D Systems, Minneapolis, MN, USA) H2AX, *γ*H2AX (both from Abcam, Cambridge, MA, USA), phospho-STAT3^Tyr705^, STAT3 (both from Cell Signaling Technologies, Danvers, MA, USA) and actin (Sigma, St. Louis, MO, USA). The band intensity was measured using ImageJ software; the values were normalized to loading control visualized by stain-free gels (Bio-Rad, Inc., Hercules, CA, USA) and presented as a fold change over 0 h time point. Experiments were performed three times.

### Statistical analysis

Kaplan–Meyer survival curves were analyzed using log-rank test. Other data were analyzed using Student's *t*-test for comparisons of two groups or one-way ANOVA and Holm–Sidak post test for multiple comparisons. All statistical tests were two sided and statistical analysis was done with the use of SigmaStat software (Systat Software Inc., San Jose, CA, USA). The differences were considered significant when *P*<0.05. Data are presented as means±S.E.M.

## Abbreviations

DDR: DNA damage response
GI: gastrointestinal
Gy: Gray
IR: irradiation
KO: knockout
PM: pyridoxamine
ROS: reactive oxygen species
SEM: standard error of the mean
WBI: whole body irradiation
WT: wild type

## Notes

PAV has the research grant support from NephroGenex, Inc. EMY, TM, WGY and AVI declare no potential conflict of interest.
